# Antibiotics in Mucogingival Surgery for Recession Treatment: A Narrative Review

**DOI:** 10.3390/antibiotics14080769

**Published:** 2025-07-30

**Authors:** Magdalena Latkowska-Wiśniewska, Sylwia Jakubowska, Bartłomiej Górski

**Affiliations:** Department of Periodontology and Oral Mucosa Diseases, Medical University of Warsaw, Binieckiego 6 St., 02-097 Warsaw, Poland; sylwiajakubowska999@interia.pl (S.J.); bgorski@wum.edu.pl (B.G.)

**Keywords:** gingival recession, mucogingival surgery, antibiotic prophylaxis, drug resistance, bacterial, surgical wound infection, wound healing, connective tissue grafts, anti-bacterial agents

## Abstract

Gingival recession is a common problem, particularly affecting oral health and esthetics, and its treatment involves surgical root coverage procedures. The aim of this narrative review is to evaluate the role of systemic antibiotic therapy in mucogingival surgery for recession treatment. The available literature does not support routine antibiotic use in systemically healthy patients undergoing recession coverage surgery. Indications for prophylactic antibiotics are restricted to individuals at high risk of infective endocarditis and immunocompromised patients with elevated susceptibility to surgical site infections. Although mucogingival surgeries are performed in a non-sterile environment, the risk of infection remains low when proper aseptic techniques and good preoperative tissue preparation are applied. The review emphasizes the importance of making clinical decisions that consider the patient’s health status and are aligned with current recommendations. It also emphasizes the necessity for prospective studies to evaluate antibiotics’ effect on recession coverage procedures outcome. To bridge the gap between contemporary evidence and clinical practice and to foster responsible use of antibiotics in periodontal plastic surgery, the authors of this review integrate current evidence and clinical guidelines into a practical tool designed to assist clinicians in making reasoned, evidence-based decisions.

## 1. Introduction

Gingival recession (GR) is defined as an apical shift in the gingival margin relative to the cemento–enamel junction (CEJ), resulting in the exposure of the root surface [1]. Gingival recession is a common condition across all age groups, with its prevalence increasing with age. According to epidemiological studies, more than two-thirds of the world’s population are affected by gingival recession [2].

Treatment of gingival recession involves surgical root coverage. The most commonly used methods include the coronally advanced flap (CAF), laterally positioned flaps, and tunnel techniques. The autogenous subepithelial connective tissue graft (SCTG), harvested from the palate, is considered the gold standard due to its well-documented clinical efficacy in gingival augmentation [3]. Surgical techniques using free gingival grafts (FGGs) are also employed in clinical practice [4].

Besides selecting the appropriate surgical procedure, identifying the cause of recession is essential for successful treatment and to avoid complications. Gingival recession has a multifactorial etiology, involving both inflammatory and non-inflammatory processes, and may present in patients with either poor or good oral hygiene [5,6,7].

Gingival recessions may occur as a clinical sign of periodontitis, a microbially induced, host-modulated inflammatory disease resulting in loss of periodontal attachment [5]. Identifying periodontitis as the underlying cause is critically important, as the main goal of treatment in such cases is to achieve remission of inflammation [8]. Only after inflammation has been resolved can appropriate planning of mucogingival surgery be undertaken.

In contrast, gingival recession developing in the absence of inflammation is often associated with alveolar bone dehiscence, which may have an anatomical origin—particularly in individuals with a thin periodontal phenotype [9]. Furthermore, recession can result from teeth being improperly positioned in their sockets, as seen in some malocclusion cases, or due to excessive buccal or lingual root movement during orthodontic treatment [10,11]. Traumatic tooth brushing, especially when excessive force is applied, is another contributing factor [12]. Moreover, muscle and frenal attachments located near the gingival margin can accelerate recession progression [13]. Although this pathway is non-inflammatory in nature, impaired plaque control on exposed root surfaces can cause plaque-induced gingivitis, increasing the risk of further gingival recession [14].

Root coverage procedures are elective interventions, allowing sufficient time for accurate diagnosis and appropriate patient preparation—an essential step that should never be omitted. Oral antibiotic therapy should never replace local anti-inflammatory treatment, regardless of whether gingival recessions result from tissue destruction due to periodontitis or if plaque-induced gingivitis has developed secondarily in previously non-inflammatory recessions.

The routine use of systemic antibiotics in root coverage procedures is not supported by current scientific evidence. Indications for systemic antibiotic administration are primarily limited to preventing surgical site infections in immunocompromised patients and for prophylaxis of infective endocarditis in individuals at high risk of this complication [15,16,17].

Surgical site infections (SSIs) can complicate any surgery, including recession coverage procedures, even in systemically healthy patients. In periodontal soft tissue surgery, SSIs may cause delayed wound healing, failure to achieve anticipated clinical outcomes, and in some cases, necessitate additional surgical intervention. Risk factors for SSIs in the preoperative, intraoperative, and postoperative periods should be identified to minimize their occurrence [18].

Considering the increasing risk of antimicrobial resistance, the rational and judicious use of antibiotics has become essential. The purpose of this article is to synthesize and systematize current knowledge to help clinicians base their therapies on scientific evidence and better plan root coverage procedures. The decision to use systemic antibiotic therapy in a recession coverage procedure should not be based solely on clinical prudence but should always be grounded in statistically proven necessity and aligned with current recommendations. To the best of the author’s knowledge, this is the first narrative review specifically aimed at organizing and summarizing the available evidence regarding antibiotic use in mucogingival surgery for the treatment of gingival recession.

## 2. Materials and Methods

Although this article is a narrative review, we followed a structured approach to ensure that the information gained is comprehensive and balanced. The literature was identified through a manual search of PubMed using combinations of Mesh Keywords such as “gingival recession”, “mucogingival surgery”, “antibiotic prophylaxis”, “drug resistance, bacterial”, “surgical wound infection”, “wound healing”, “connective tissue grafts”, and “anti-bacterial agents”. Additionally, reference lists of selected articles were examined, and the “Related Citations” feature in PubMed was used to identify further related studies. The review focused on peer-reviewed clinical studies, systematic reviews, narrative reviews, randomized controlled trials, and international guidelines related to antibiotic use in mucogingival surgery. Relevant scientific textbooks were also included to provide foundational knowledge. Case reports were excluded from the search.

All searches focused on articles published in English within the past ten years; however, older articles found through reference lists were included when relevant to the review.

## 3. Defining Indications for Systemic Antibiotics in Mucogingival Surgery for the Treatment of Gingival Recession

The recession coverage procedure can be classified as a clean-contaminated wound surgery. Oral procedures, even when performed under aseptic conditions, come in contact with the natural bacterial flora of the oral cavity [18]. Administration of antibiotics may serve either as prophylaxis, aimed at preventing surgical site infections or distant infections, or as therapy for an existing infection originating from the healing wound following a recession coverage procedure.

Antibiotic prophylaxis has two main purposes: to prevent surgical site infections and to prevent distant infection. The decision to begin prophylactic antibiotic therapy should be based on careful evaluation of the patient’s history, the risk for infection, and the associated risk factors related to the nature of the surgical procedure. This decision must be made in accordance with current evidence-based clinical guidelines and recommendations. It should be emphasized that the optimal timing for preoperative surgical prophylaxis is always before the surgical incision.

Prophylaxis aimed at preventing distant infections is indicated for patients at the highest risk of developing infective endocarditis, with oral streptococci being the primary microbial agents targeted by such interventions [16,17].

Prophylaxis to prevent surgical site infections should be considered in immunocompromised patients. These individuals may be more susceptible to postoperative complications due to impaired immune response [19].

When assessing intraoperative risk for surgical site infection during a recession coverage procedure, several factors should be considered, such as the naturally contaminated environment of the oral cavity [18], the expected length of the procedure [20], and the particular healing architecture of the involved tissues. Healing is particularly susceptible to ischemia due to the fact that both the flap and the graft come into contact with the avascular root surface [21].

It should be strongly underlined that, as part of infection prevention in dentistry, the maintenance of proper oral hygiene and strict adherence to principles of asepsis and antisepsis during perioperative care are of fundamental importance. These measures remain the cornerstone of effective infection control in dental procedures [18].

### 3.1. Antibiotic Prophylaxis in Patients at High Risk of Infective Endocarditis

Infective endocarditis (IE) is a serious issue of public health. In 2019, the global incidence was estimated at 13.8 cases per 100,000 people per year, with approximately 66,300 deaths occurring worldwide as a result of the disease [22]. Given the substantial morbidity and mortality associated with infective endocarditis, identifying effective preventive strategies is critical [22,23].

The oral cavity hosts a significant population of commensal microorganisms, including oral streptococci, and serves as a major entry point for pathogens. Dental procedures—including tooth extractions, periodontal and implant surgery, and oral biopsies—or any manipulation of the gingival tissue or the periapical region of teeth, are thought to be high-risk procedures for the induction of bacteremia. This risk also applies to mucogingival surgery for the treatment of gingival recession [24,25,26,27].

The recommendation for antibiotic prophylaxis for patients at moderate and high risk of infective endocarditis has been supported by population-based studies, where efficacy was assessed in terms of bacteremia as surrogate marker for IE [28,29,30,31,32]. Further evidence is provided by several animal and observational studies [28,33,34,35,36,37,38,39,40,41,42].

**Figure 1:**
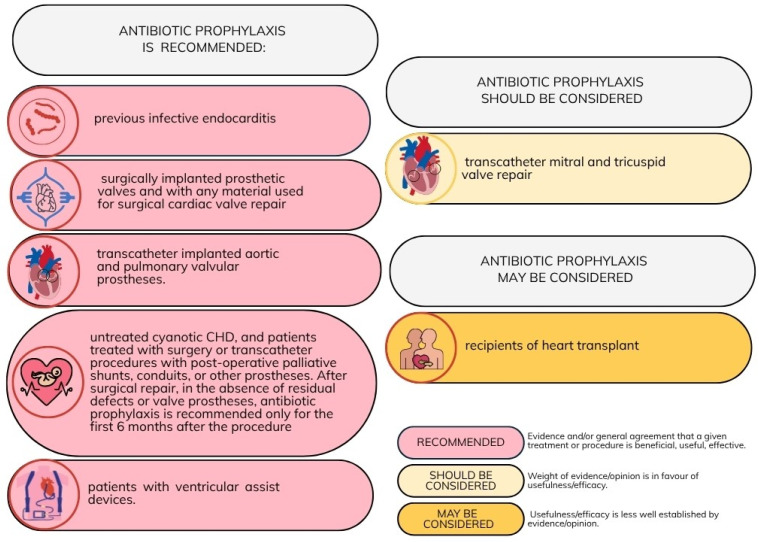
Recommendations for antibiotic prophylaxis in patients with cardiovascular diseases undergoing oro-dental procedures at increased risk for infective endocarditis [16]. Adapted with permission from Ref. [16]. Copyright 2023, Oxford University Press.

The latest recommendations for antibiotic prophylaxis of oro-dental procedures in CVD patients were published in 2023 by the ESC [16]. These guidelines list patient groups at high risk of infective endocarditis, in whom antibiotic prophylaxis is either recommended or may be considered. These risk groups are outlined in Figure 1.

**Figure 2:**
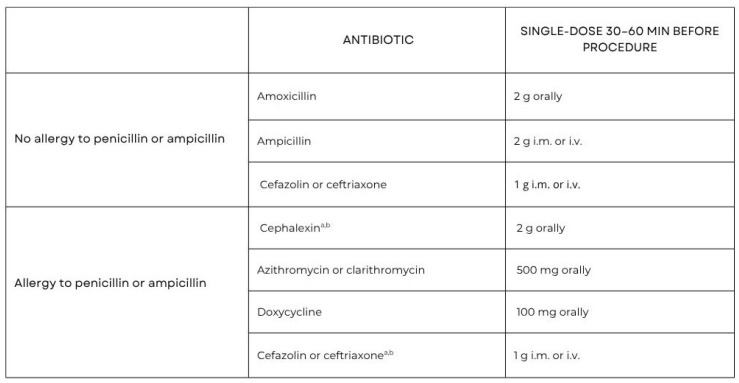
Prophylactic antibiotic regime for IE high-risk dental procedures and doses for adults [16,17]. a—Other first- or second-generation oral cephalosporin in equivalent dosing; b—Cephalosporins should not be used in an individual with a history of anaphylaxis, angioedema, or urticarial with penicillin or ampicillin. Adapted with permission from Ref. [16]. Copyright 2023, Oxford University Press.

A single oral dose of 2 g of amoxicillin without clavulanic acid is recommended to be administered prior to the surgical procedure. In patients with a penicillin allergy, cephalexin, azithromycin, clarithromycin, or doxycycline may be used as alternative agents. Importantly, clindamycin is no longer recommended for antibiotic prophylaxis because of a higher risk of severe adverse events, including fatal outcomes, mainly associated with *Clostridioides difficile* infections [16,17]. Figure 2 summarizes the main antibiotic prophylaxis regimens recommended before dental procedures, including intravenous administration routes where applicable.

It should be emphasized that, according to both the 2023 European Society of Cardiology (ESC) guidelines [16] and the earlier 2021 Scientific Statement from the American Heart Association [17], general prevention measures—such as maintaining good oral hygiene, professional dental cleaning, and follow-up at least twice a year—are of key importance.

In cases where surgical procedures are required in these patients, in addition to antibiotic prophylaxis, all strategies aimed at minimizing the risk of surgical site infection should be implemented.

This is of special significance for patients with periodontitis where a lack of active inflammation is a mandatory requirement before performing any intended periodontal surgical procedure [37].

### 3.2. The Specific Nature of Recession Coverage Procedures in the Context of Potential Postoperative Infection

Surgical interventions, including recession coverage procedures, are associated with a potential risk of postoperative complications. Since these procedures are carried out in the contaminated environment of the oral cavity, there is a possibility of wound infection—either due to the oral conditions themselves or as a result of flap stabilization and suturing methods [18].

Most recession coverage methods result in healing by primary intention [18,43,44]. The aim of the procedure is to lengthen the flap in order to restore soft tissue architecture, precisely approximate the wound margins, and secure them with sutures. Additionally, a subepithelial connective tissue graft (SCTG), harvested from the palatal mucosa, is commonly employed beneath the flap. This graft serves to enhance soft tissue thickness, provide mechanical support for flap stabilization, and promote optimal healing conditions by facilitating clot stability and integration with the recipient site [4].

Healing by primary intention creates conditions for the formation of a stable clot, shortens the duration of the inflammatory phase of healing, and reduces the risk of bacterial contamination of the wound [18,43].

However, in the event of postoperative infection, the enclosed nature of the wound may prevent adequate drainage, allowing bacterial proliferation and the accumulation of degradation products within the tissue, ultimately leading to abscess formation. The resulting increase in intrawound pressure can impair local perfusion, further exacerbating the spread of infection. This sequence of events may culminate in wound dehiscence, which, although it permits evacuation of purulent material, concurrently compromises the surgical outcome [43].

**Figure 3:**
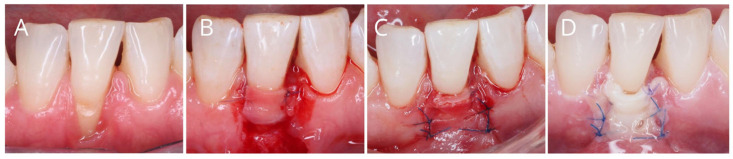
(**A**–**C**), Performance of periodontal plastic surgery (coronally advanced flap plus connective tissue graft) in mandibular incisor affected by gingival recession. (**D**), Flap necrosis one week after surgery.

The healing process following a recession coverage procedure is particularly susceptible to ischemia, as both the flap and the graft come into contact with the avascular root surface [21]. This creates a risk of tissue necrosis, defined as the irreversible death of body tissue resulting from injury, radiation, or chemical exposure [45]. In the context of recession coverage surgery, necrosis may involve the flap, a free gingival graft, or subepithelial connective tissue, especially when bilaminar techniques are employed. Necrotic tissue can behave as a foreign body, potentially leading to a purulent exacerbation during the healing phase [18] (Figure 3).

In periodontal soft tissue surgery, surgical site infection may lead to prolonged healing, failure to achieve the expected clinical benefits of the intervention, and the need for second surgical intervention. Fortunately, available evidence confirms that the risk of surgical site infection following recession coverage procedures is low.

Based on retrospective study by Askar et al. (2019) [46], procedures involving connective tissue grafts (CTGs) demonstrated a low incidence of postoperative complications, most of which were mild in nature. Surgical site infection occurred only in 0.3% of cases. The most common complication was excessive pain, reported in 3.3% of patients, followed by delayed wound healing in 2.3% of cases. Mild postoperative bleeding and flap necrosis were each observed in 2% of patients.

According to Powell et al. (2005) [47], the incidence of SSI following procedures involving subepithelial connective tissue autografts was 3.66% (3 out of 82 patients).

Due to limited data on reported morbidity, the recent Cochrane review by Chambrone et al. [48] did not include specific figures on the frequency of complications following root coverage procedures. According to this review, the adverse effects associated with root coverage treatments for localized and multiple recession defects were mainly discomfort and pain, typically linked to the donor site where the graft was taken. These symptoms usually appeared within the first week after the surgery and did not affect the overall success of the root coverage [48].

Regarding the donor site of subepithelial tissue grafts, Harris et al. [49] reported a donor site infection rate of 0.8%, with only one infection occurring in 500 cases; the infection was located at the sutures of the palatal wound [46]. A retrospective analysis by Aguirre-Zorzano et al. (2017) found a 7.5% rate of donor site infections following the harvesting of subepithelial connective tissue grafts, with three infections observed among 40 cases [50].

### 3.3. Identification of Patients at Higher Risk of Surgical Site Infection and Decision-Making Regarding Antibiotic Therapy

Each of the various functional compartments of the immune system contributes uniquely to host defense, and dysfunction in a specific compartment can markedly increase the risk of SSIs. Immunodeficiencies can be divided into primary and secondary immunodeficiencies. Primary immunodeficiencies involve congenital immune deficiencies. Secondary immunodeficiencies are those that are related to another illness or condition or occur as a result of treatment for such a condition [51,52].

To ensure the proper diagnosis of a systemic disease potentially affecting immune system function, a thorough patient history must always be obtained. Additionally, consultation with the physician managing the underlying disease is essential to assess the suitability for planned periodontal procedures and to determine the safest form of antibiotic prophylaxis. This prophylaxis often needs to be individualized based on the patient’s overall health status. Antimicrobials should be given within 1 h prior to the surgical incision, except for vancomycin and fluoroquinolones, which should be administered within 2 h before the incision.

Diabetes mellitus is one of the most prevalent chronic disorders associated with a higher risk of surgical site infection (SSI). Perioperative antibiotic prophylaxis should be considered for these patients. Neutrophils from diabetic patients demonstrate impaired chemotaxis and a diminished ability to kill bacteria via oxidative mechanisms compared to non-diabetic individuals. Such dysfunction may favor bacteria for growth and may negatively modulate fibroblast function and collagen synthesis, as well as affect wound healing. In the context of planned surgical procedures, micro- and macroangiopathies present an additional challenge by reducing tissue perfusion, thereby increasing the risk of necrosis [53]. Since glycosylated hemoglobin (HbA1c) reflects long-term glycemic control, it has been suggested that achieving optimal preoperative glycemic levels (HbA1c below 7%) may lead to a lower rate of postoperative infections [20].

Smoking, older age, low serum albumin concentration, and ischemia secondary to vascular disease or irradiation are also recognized risk factors for surgical site infection [54]. Given that recession coverage procedures are elective in nature, patients should always be encouraged to cease smoking prior to surgery [55]. It is also advisable to check the serum albumin level prior to the procedure. In patients with a history of head and neck radiotherapy, periodontal treatment should mainly consist of non-surgical therapy and prophylaxis, including fluoride application to exposed root surfaces. Considering the increased risk of osteoradionecrosis, electively mucogingival surgery should be avoided in these patients [56].

**Figure 4:**
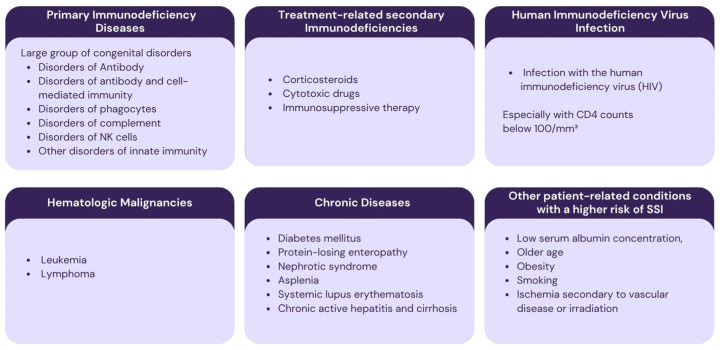
Patients at higher risk of developing surgical site infection.

Patients at higher risk of developing SSI are presented in Figure 4.

### 3.4. Antibiotic Prophylaxis for Surgical Site Infection in Systemically Healthy Patients

The body of research examining the impact of antibiotic therapy on the outcomes of mucogingival surgery for recession treatment remains limited. Moreover, there is a lack of prospective studies specifically evaluating the influence of antibiotic therapy on the clinical outcomes of such procedures.

Based on retrospective study by Askar et al. (2019) [46], the use of antibiotics was associated with a significant reduction in the incidence of delayed wound healing (*p* = 0.049). Although it highlights the potential benefits of antibiotics in postoperative management, given the long time span during which the procedures were performed (1990–2018) and the evolution of surgical techniques for recession coverage over this period, the findings should be interpreted with caution. What is more, all the included procedures were performed within an academic setting by graduate students with expert faculty supervision, potentially increasing the number of complications.

According to Powell et al. (2005) [47], in a retrospective review, patients who received antibiotics as part of the surgical protocol for various periodontal procedures, including mucogingival surgeries (pre- and/or postoperatively), developed surgical site infections (SSIs) in 8 out of 281 procedures (2.85%). In contrast, 14 infections occurred in 772 procedures (1.81%) where antibiotics were not administered; however, this difference was not statistically significant.

According to Pack and Haber (1983) [57], the overall incidence of postoperative infection following periodontal surgery was 1% (9 out of 927 procedures). No significant difference was observed between patients who received prophylactic antibiotic therapy and those who did not. Specifically, among the 43 procedures performed with antibiotic prophylaxis, 1 postoperative infection occurred (2%), while 8 infections were recorded among the 884 procedures performed without prophylactic antibiotics (0.9%). All infections responded well to therapeutic antibiotic treatment and resolved within 48 to 72 h. Based on these findings, the authors concluded that in the absence of specific medical indications, the routine use of prophylactic antibiotic therapy to prevent postoperative infection in periodontal surgery is not justified.

According to Mazzotti et al. (2023) [21] as well as Zuhr and Hürzeler (2012) [43], antibiotic therapy should be recommended only during the acute phase. When wound dehiscence occurs, they recommend daily follow-up visits with irrigation of the wound using a 0.1% chlorhexidine solution and the local application of chlorhexidine gel. These measures should be continued until complete healing is achieved. In the case of abscess formation, an attempt should be made to drain it through the gingival pocket. If this is not possible, the abscess should be incised under local anesthesia, the abscess cavity be irrigated with a 0.1% chlorhexidine solution, and passive drainage should be applied, along with daily follow-up visits until complete healing.

Oral antibiotic therapy is recommended in cases where the infection spreads or the patient’s general condition worsens (the occurrence of low-grade or high fever, malaise, tachycardia). The canine fossa is an area that requires mandatory systemic antibiotic therapy in cases of purulent exacerbation, due to the risk of infection spreading intracranially [43].

Although systemic antibiotics have a role in managing purulent exacerbations during the healing phase after mucogingival procedures, it is important to emphasize that systemic antibiotics cannot substitute for proper asepsis, antisepsis, and preoperative optimization of local tissue health. The local reduction in bacterial load—both prior to surgery and throughout the healing period—should remain the primary focus in preventing postoperative infections and ensuring successful outcomes.

## 4. Asepsis, Antisepsis, and Preoperative Optimization of Local Tissue Health

**Figure 5:**
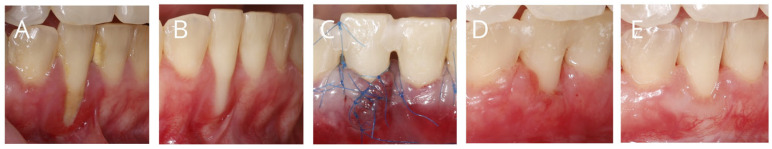
(**A**), Plaque-induced gingival inflammation as a contraindication for performing a surgical recession coverage procedure; (**B**), the clinical situation, following improvement in the patient’s plaque control and professional mechanical plaque-removal procedures; (**C**), recession coverage with laterally closed tunnel and SCTG; (**D**), 2 weeks post-surgery; (**E**), 6 months post-surgery.

Before performing a recession coverage procedure, inflammation of the gingiva and periodontium should be eliminated, and an adequate level of oral hygiene must be achieved. (Figure 5). The full-mouth bleeding on probing (BOP) should be below 20%. This is extremely important, as inflamed tissues are more susceptible to damage and perforation during surgery. What is more, surgical procedures performed on inflamed tissues are associated with an increased risk of surgical site infection [18,20].

It is recommended that, prior to the procedure, the patient rinse their mouth with a 0.2% chlorhexidine solution to reduce the bacterial load in the oral cavity. All teeth should be professionally debrided, and any particles that may act as foreign bodies, such as calculus, should be removed before making the incision [18,21].

During the procedure, strict aseptic techniques must be maintained to prevent the introduction of microorganisms from hands, surfaces, or equipment into the sterile surgical area. This is especially important for mucogingival procedures, which often last several hours. Aseptic techniques include surgical hand scrubbing, the use of sterile gloves, sterile gowns, and sterile surgical drapes. Prior to surgery, antiseptic skin preparation with an alcohol-based chlorhexidine solution should be performed. If hair removal is necessary, the patient should be informed beforehand and advised to use an electric clipper instead of a razor [20,45,58].

During the procedure, regular irrigation of the surgical field with saline is good practice. Frequent postoperative check-ups are essential to detect early signs of impaired healing, wound dehiscence, or potential infection.

A specific recommendation for recession coverage procedures is that patients must refrain from brushing the operated area until suture removal. Mechanical plaque control must be replaced by chemical control during this time. This aims to reduce the bacterial count, which is extremely important for proper wound healing. The bacterial count around the wound is a reliable indicator of whether an infection will occur. Wounds that contain more than 100,000 bacteria per gram of tissue are likely to become infected. On the other hand, wounds with lower bacterial count generally heal properly without infection [45].

To ensure proper plaque control and reduce the infectious burden in the oral cavity during healing, it is recommended to rinse the mouth with a 0.1–0.2% chlorhexidine solution and to apply chlorhexidine gel directly to the wound site. Chlorhexidine is capable of reducing plaque formation by 30–80%, and its use is well established, making it the gold standard for postoperative care and prevention of surgical site infections [18,59,60,61,62].

## 5. Discussion

There is no doubt that the principal aim of the surgical operator is to employ all possible strategies to prevent the occurrence of surgical site infections and to ensure uncomplicated wound healing as well as the patient’s well-being during the postoperative period. This may contribute to the overprescription of antibiotics and their use in otherwise healthy patients undergoing procedures where the risk of surgical site infection (SSI) is relatively low.

Excessive antibiotic prescribing carries a significant risk of developing antibiotic resistance (AMR). Without urgent and coordinated action, AMR threatens to reverse decades of progress in medicine, making once-treatable infections potentially deadly again. Projections suggest a 70% rise in deaths attributable to AMR by 2050 if current trends persist. Contributing factors include aging populations, increased comorbidities such as obesity and diabetes, and continued antibiotic overuse in both human medicine and agriculture. Key strategies to combat AMR include antibiotic stewardship programs (ASPs) and infection prevention and control [63,64,65,66,67].

According to current recommendations, antibiotic prophylaxis before a recession coverage procedure applies to patients in the high-risk group for infectious endocarditis and immunocompromised patients with an increased risk of surgical site infection. In both cases, it should be prescribed prior to the procedure and given as a one-shot dose [15,16,17].

Most available studies do not confirm clinical benefits of antibiotic prophylaxis in generally healthy patients undergoing recession coverage, limiting the use of antibiotics to cases of acute conditions [21,43,47,57]. Notably, other studies investigating the effect of antibiotic prophylaxis on the risk of implant failure and the incidence of postoperative complications after tooth extraction in systemically healthy patients have also reported no statistically significant differences [68,69].

It should be remembered that prevention of infection at the surgical site should primarily involve patient preparation before the procedure, including elimination of gingival inflammation, proper aseptic and antiseptic techniques, and chemical plaque control during healing. Regular follow-up visits during the healing process are also extremely important [18,20,21,45,58,59,60,61,62].

Conducting prospective studies on the impact of antibiotic prophylaxis on the outcomes of recession coverage procedures is a promising direction for future research.

## 6. Limitations

It is important to note that, unlike systematic reviews—which follow established frameworks such as the PRISMA guidelines—narrative reviews are more susceptible to subjective interpretation and selective reporting.

One key limitation of this narrative review is the lack of prospective studies examining the impact of antibiotic prophylaxis on the outcomes of recession coverage procedures. Furthermore, the number of available retrospective studies evaluating the role of antibiotic therapy in the results of recession coverage surgery is limited. This may affect the reliability of the conclusions. Therefore, the findings should be regarded as informative but interpreted with caution.

## 7. Conclusions

The majority of studies fail to demonstrate clinical benefits of antibiotic prophylaxis in healthy patients undergoing mucogingival surgeries; however, the number of studies on this topic is limited. Current guidelines recommend antibiotic prophylaxis before recession coverage procedures only for patients at high risk of infectious endocarditis and for immunocompromised individuals with an increased risk of surgical site infections. There is a lack of prospective studies evaluating the impact of antibiotic therapy on the clinical outcomes of recession coverage procedures. Based on available retrospective studies, the risk of surgical site infection in recession coverage procedures is low (below 3.66%), which undermines the rationale for prophylactic systemic antibiotic therapy in otherwise healthy patients. Individualized clinical judgment taking into account the patient’s overall health status is essential. The optimization of tissue health preoperatively and a strict adherence to aseptic and antiseptic principles for the prevention of surgical site infections are also of paramount importance.

## Data Availability

No new data were created or analyzed in this study. Data sharing is not applicable.

## Abbreviations

The following abbreviations are used in this manuscript:

STI	Surgical site infection
CVD	Cardiovascular disease patients
CAF	Coronally advanced flap
SCTG	Subepithelial connective tissue graft
